# Supplementary material for: Partial Agonist Activity of Neonicotinoids on Rat Nicotinic Receptors: Consequences over Epinephrine Secretion and In Vivo Blood Pressure
Source: Int J Mol Sci. 2021 May 12;22(10):5106. doi: 10.3390/ijms22105106 (PMC8151892; doi:10.3390/ijms22105106)
Supplement: Supplementary file 1 [file ijms-22-05106-s001.zip › ijms-1183743-supplementary.pdf]

nAChRs (species)	Expression system	Assay	NIC	ACE	CLO	IMI	Agonist effect	References
α3β4 (rat)	Xenopus oocyte	Membrane current	EC_{50} = 4.62 μM	EC_{50} = 0.13 M	EC_{50} = 8.39 mM	n/a	weak	This study
α4β2 (human)	HEK 293	Membrane current	n/a	n/a	EC_{50} ~ 0.100 mM	EC_{50} ~ 0.05 mM	weak	Li *et al.*, 2011 (41)
α7 (rat)	Xenopus oocyte	Membrane current	n/a	EC_{50} = 0.74 mM	EC_{50} = 0.74 mM	n/a	strong	Cartereau *et al.*, 2018 (42)

n/a: not available.

Table S1

nAChR subunit	Primer designation	Primer sequence (5’-3’)[a]	Purpose
α3	nAChSA	GCACCAGAGTGTCTCCCTCCC	Full-length cDNA
	nAChRB	AGCCTCGATGAACAGTGCACAG	
	nAChS1	ttaaaagaattcgccaccATGGGTGTTGTGCTGCTCCC	pGEMHE-ORF
	nAChR2	tgatataagcttCTATGTGTCATCTCTGGCCATCA	
β4	nAChSC	GGCTGCCACCCGGCTGGCC	Full-length cDNA
	nAChRD	ACGCCGGGTAGCCTAGGAGTC	
	nAChS3	ttaaaagaattcgccaccATGAGGGGTACGCCCCTG	pGEMHE-ORF
	nAChR4	tgatataagcttCTAGGAGTCCTTGGAGGGTGC	

[a]The restriction sites and Kozak sequence are indicated by single (EcoRI; HindIII) and double underlining, respectively.

Table S2

nAChR subunit	Nucleotide variation (CNS *vs* AM)	Location[a]	Amino-acid modification
α3	T816C	M2	-
	C903T	M3	-
	G1011A	M3-M4 intracellular loop	-
	G1102A	M3-M4 intracellular loop	G368S
β4	T411C	E loop	-
	G1261C	M3-M4 intracellular loop	G421R

[a] Functional miscellaneous features of each nAChR subunit transcript are extracellular loops (A to F loops) involved in the ACh binding site and transmembrane domains (M1 to M4) linked by intracellular and extracellular loops and forming the channel pore.

Table S3

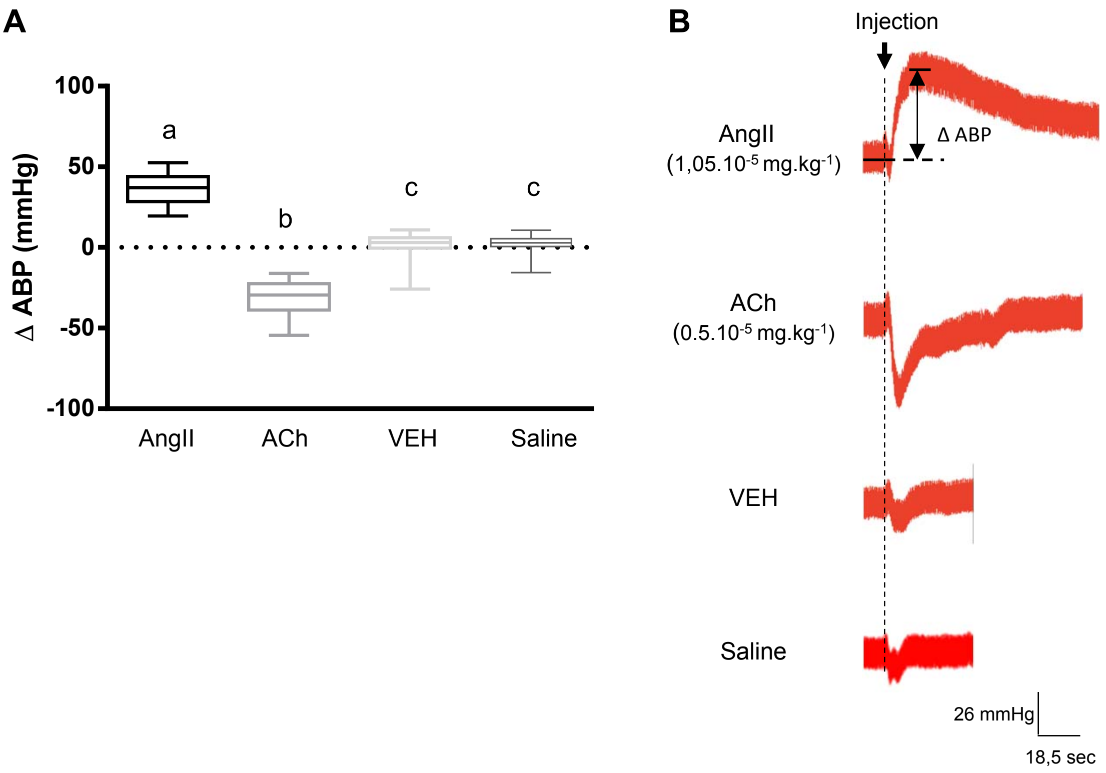
A
100
50
0
-50
-100
Δ ABP (mmHg)
a
b
c
c
AngII
ACh
VEH
Saline
B
Injection
AngII
(1,05.10-5 mg.kg-1)
Δ ABP
ACh
(0.5.10-5 mg.kg-1)
VEH
Saline
26 mmHg
18,5 sec

Figure S1

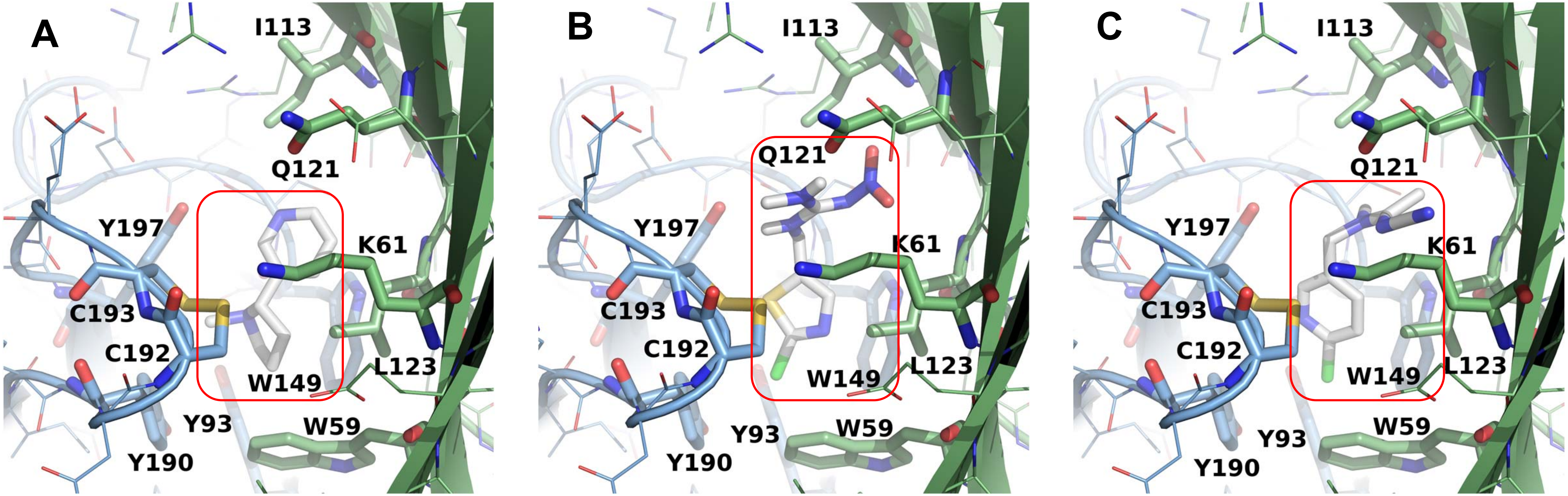
A
I113
Q121
Y197
K61
C193
C192
L123
W149
Y93
W59
Y190
B
I113
Q121
Y197
K61
C193
C192
L123
W149
Y93
W59
Y190
C
I113
Q121
Y197
K61
C193
C192
L123
W149
Y93
W59
Y190

Figure S2

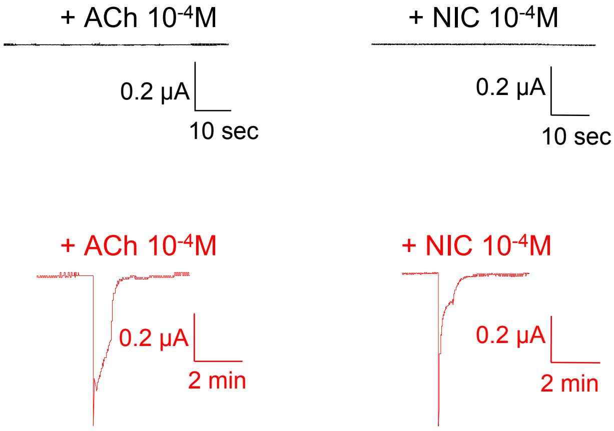

+ ACh 10-4M
+ NIC 10-4M
0.2 μA
10 sec
0.2 μA
10 sec
+ ACh 10-4M
+ NIC 10-4M
0.2 μA
2 min
0.2 μA
2 min

Figure S3

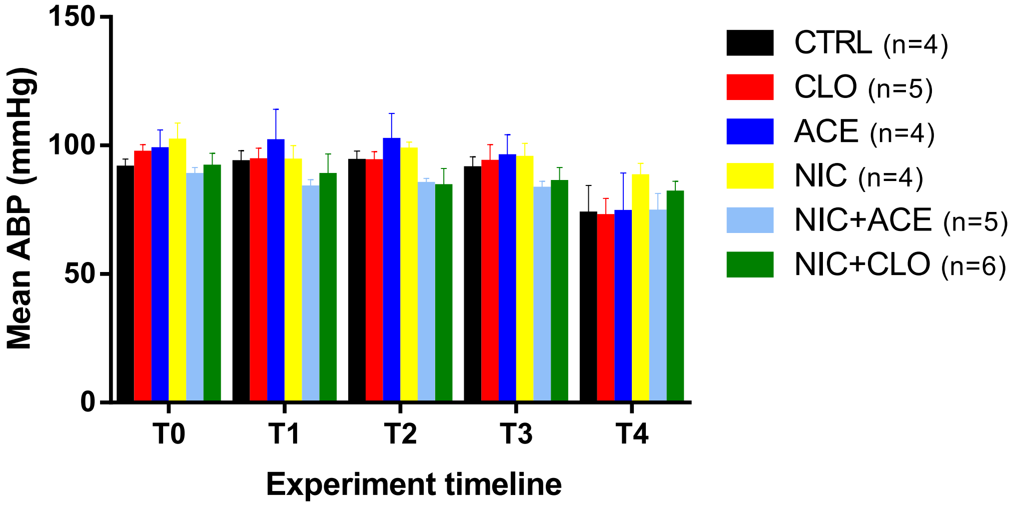
150
100
50
0
Mean ABP (mmHg)
T0
T1
T2
T3
T4
Experiment timeline
CTRL (n=4)
CLO (n=5)
ACE (n=4)
NIC (n=4)
NIC+ACE (n=5)
NIC+CLO (n=6)

Figure S4
